# Supplementary material for: Intrinsic and extrinsic pinning in NdFeAs(O,F): vortex trapping and lock-in by the layered structure
Source: Sci Rep. 2016 Oct 26;6:36047. doi: 10.1038/srep36047 (PMC5080545; doi:10.1038/srep36047)
Supplement: Supplementary Information [file srep36047-s1.pdf]

SUPPLEMENTARY INFORMATION FOR: Intrinsic and extrinsic pinning in NdFeAs(O,F): vortex trapping and lock-in by the layered structure

C. Tarantini,[1*] K. Iida,[2,3] J. Hänisch,[2,4] F. Kurth,[2,5] J. Jaroszynski,[1] N. Sumiya,[3] M. Chihara,[3] T. Hatano,[3] H. Ikuta,[3] S. Schmidt,[6] P. Seidel,[6] B. Holzapfel,[4] D.C. Larbalestier[1]

[1] Applied Superconductivity Center, National High Magnetic Field Laboratory, Florida State University, Tallahassee FL 32310, USA

[2] Institute for Metallic Materials, IFW Dresden, 01171 Dresden, Germany

[3] Department of Crystalline Materials Science, Nagoya University, Chikusa-ku, Nagoya 464-8603, Japan

[4] Institute for Technical Physics, Karlsruhe Institute of Technology, 76344 Eggenstein-Leopoldshafen, Germany

[5] Dresden University of Technology, Faculty for Natural Science and Mathematics, 01062 Dresden, Germany

[6] Friedrich-Schiller-University Jena, Institute of Solid State Physics, 07743 Jena, Germany

The NdFeAs(O,F) thin film was characterized by XRD. The θ-2θ scan in Fig. S1(a) shows only the 00l reflections of the NdFeAs(O,F) phase. The 003 rocking curve has a narrow full width at half maximum (FWHM) of $\Delta\omega$ = 0.62° [Fig. S1(b)]. Both demonstrate an excellent out-of-plane orientation. The 102 ϕ-scan of NdFeAs(O,F) in Fig. S1(c) exhibits a sharp FWHM, $\Delta\phi$ = 1.26°, and reveals a fourfold symmetry indicative of epitaxial growth with a (001)[100]NdFeAs(O,F)//(001)[100]MgO relation (cube-on-cube).

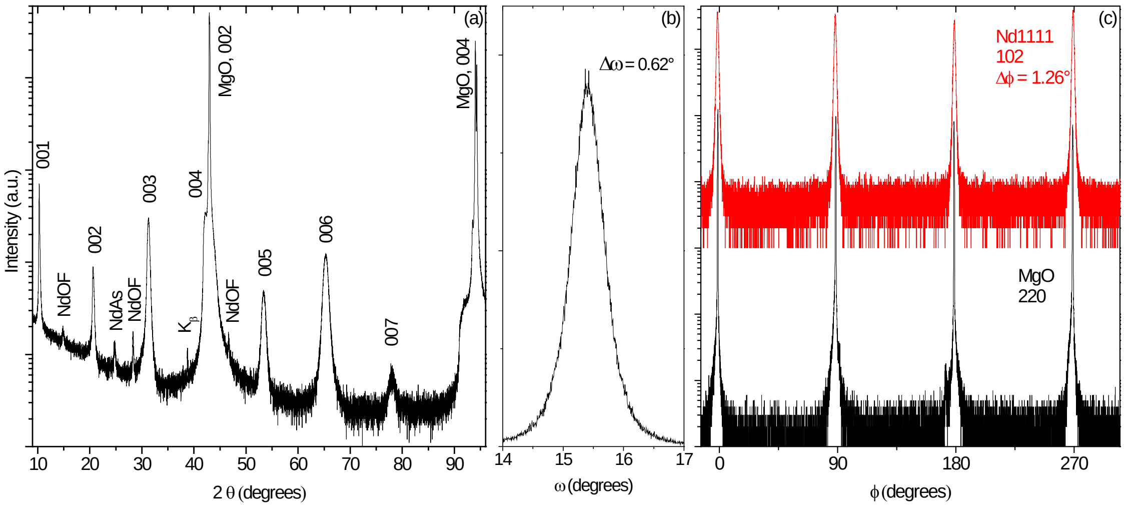

Figure S1 (a) θ-2θ scan of NdFeAs(O,F) measured after the ion beam etching. (b) Rocking curve of the (003) reflection of NdFeAs(O,F). (c) The ϕ-scans on the (102) peak of NdFeAs(O,F) thin film and on the (220) peak of MgO substrate.

The angular dependencies of J_c were measured in a wide temperature range between 4.2 and 35 K up to 35 T at 4.2 K and up to 16 T at higher temperatures. The data are reported in Fig. S2.

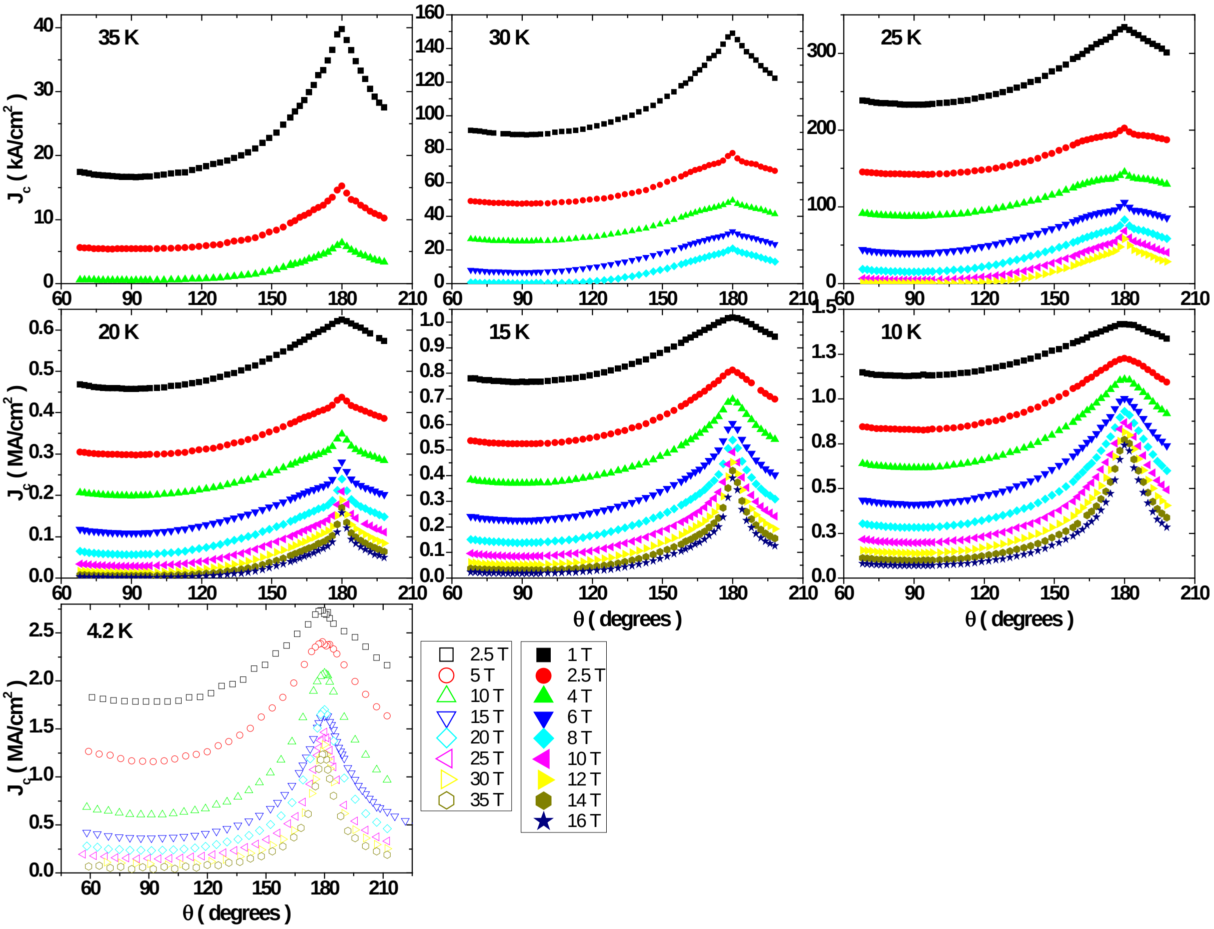

Figure S2 Angular dependence of J_c measured between 35 and 4.2 K and up to 35 T at 4.2 K, up to 16 T at higher temperature.

The *I-V* characteristics measured on the NdFeAs(O,F) thin film show a linear trend in log-log scale at every temperature, field and angular position. Typical *I-V* curves are shown in Figure S3.

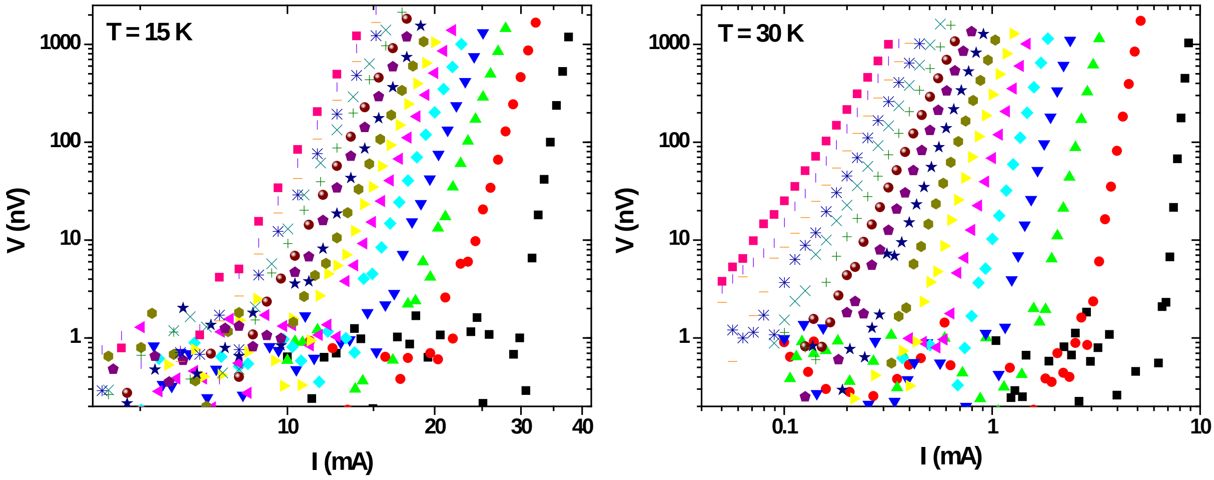

Figure S3 *I-V* characteristics at 15 and 30 K varying magnetic field from 0 to 16 T at 1 T step.
